# Experiences of clinicians and managers in the implementation of a family focused model in child and adult psychiatry

**DOI:** 10.3389/fpsyt.2024.1360375

**Published:** 2024-03-18

**Authors:** Camilla Linderborg, Anne Grant, Bente Margrethe Weimand, Adrian Farrel Falkov, Margareta Östman

**Affiliations:** ^1^ Faculty of Health, Social and Welfare Studies, USN- University of South Eastern Norway, Drammen, Norway; ^2^ Child and Adolescent Psychiatry, Stockholm Regional Council, Stockholm, Sweden; ^3^ School of Nursing and Midwifery, Queens University Belfast, Belfast, United Kingdom; ^4^ Faculty of Health and Social Sciences Mental Health and Addiction Services, Department for Research and Development, Akershus University Hospital, Oslo, Norway; ^5^ Department of Child and Youth Mental Health, Royal North Shore Hospital, Northern Sydney Health District, Sydney, NSW, Australia; ^6^Malmö University, Malmö, Sweden

**Keywords:** service development, family focused practice, collaboration, the family model, trust based process

## Abstract

**Introduction:**

This paper describes the process of implementing a family focused model, *The Family Model*, in child and adolescent and adult mental health services in Sweden. Additionally, it describes a service development project carried out in both services within a defined geographical area of Region Stockholm. *The Family Model* is a communication tool designed to assist clinicians in both services to have family focused conversations with their patients and relatives. Internationally, the needs of individuals experiencing mental health challenges (parents, children and young people) and their close relatives are now well recognized, but barriers to family focused practice nevertheless persist. The aim of this study was to better understand clinicians` experiences in implementing *The Family Model* in both services.

**Methods:**

Three preplanned focus group interviews were carried out with 14 clinicians and managers across both services and the data were analyzed in accordance with methods of Naturalistic inquiry.

**Result:**

Findings suggest that *The Family Model* has utility in both services. The Naturalistic inquiry analyses revealed three main themes: individual, relational and organizational aspects with a total of 10 sub-themes of how the models influence the participants. Furthermore, analyses on a meta understanding level explored that participants underwent a developmental journey in learning about and using *The Family Model* in practice which was expressed through three themes: “Useful for burdened families”, “Influencing prevention”, and “To integrate this would be fantastic”.

**Conclusions:**

*The Family Model*, when adapted for the Swedish context, is a useful tool for assisting experienced clinicians to engage in family focused practice in both child and adolescent and adult mental health services. *The Family Model* highlights different aspects in everyday clinical services that were of special interest for clinicians, families, and the system. Future research could explore families’ perspectives of the utility of the model.

## Introduction

An increasing number of children and adults are seeking psychiatric services for experiences of mental disorder and associated symptoms in the hope of experiencing meaning and joy in their lives again. In recent decades, psychiatric services in Sweden, as in other western countries, have offered increasingly specialized interventions (1, 2). Treatments have been increasingly based on patient diagnoses and available resources have been targeted towards clinics and units focused on individuals with the same diagnosis or experiencing similar symptom profiles. In this context of subspecialized treatments, based on individual needs, an innovative (by Swedish standards) family focused approach – *The Family Model (TFM)* (3) was implemented as part of a service development (service quality improvement) project. More than 300 families participated in the initiative. *The Family Model* targets families in which one or more members or relatives experience mental illness and who are in contact with any psychiatric services, child and adolescent mental health services (CAMHS), or adult mental health services (AMHS). *The Family Model* figure draws clinicians’ attention to the reciprocal relationship between childrens’ and parents’ mental health and general wellbeing and to the importance of focusing on the families’ strengths, protective factor, resources, and culture. Furthermore, the model highlights the various entry points for service provision for families and the need for services to work together (including joint care plans) to provide optimal support, particularly for families experiencing multiple adversities (4). The model consists of a visual illustration of six key areas (domains) and interconnecting arrows, which represent key inter-relationships between domains, with six overarching principles.

All members of a family are affected when a relative experiences mental illness (5–7), but children’s physical and mental health and development (8, 9) and social life (7, 10, 11) may be particularly impacted. Equally, parenting a child with mental health and/or physical problems can further exacerbate parental mental illness (12). Research has shown that about one-third to a half of parents of patients admitted to child psychiatric care screen positive for a psychiatric diagnosis (13–15). It is well established that family cohesion and social support are important factors for healthy development and minimizing risk factors (16–18).

A recent systematic review (19) emphasizes the value of early intervention to create health promoting and preventive measures for families at risk, especially when more than one family member experiences a mental illness. However rather than family focused interventions, western mental health services are usually offering treatment focused on the individual, despite increasing global expectations that health services tailor interventions to engage and support relatives of patients (2). There are similar expectations for improving collaboration and coordination within and between services and agencies (20–22).

A preliminary survey carried out in a Swedish setting indicated that more than half the individuals in receipt of care from CAMHS had more than one family member seeking mental health care from other services. The corresponding figure for AMHS is approximately one third of all patients (23). These findings have been replicated in a register study in Sweden (15). Many of the patients seeking psychiatric services are therefore members of families accessing multiple healthcare services. For these families, this may involve simultaneous and extensive engagement with several professionals, at a variety of specialized units. Research has shown a wish and need for greater involvement of all family members during treatment of a family member, to improve outcomes through greater awareness/knowledge, support and communication (22, 24), which has been further underlined by laws and regulations in most western countries stating that adult and children as next of kin must be included and receive necessary support. However, research has shown that healthcare personnel experience various obstacles that stand in the way of implementing family focused practice (FFP). Barriers exist at individual, relational, and organizational levels (25–28).

A number of models have been developed to guide FFP (e.g., 29–34). Such models assist in identifying points of intervention for children, parents and families and highlight the various pathways of risk for families (35). As previously noted, *TFM* has an explicit emphasis on, and illustrates, the reciprocal nature of parents’ mental illness and children’s well-being (and vice versa), and this relationship is considered a core feature of the health and illness dynamics in these families (36–38). By highlighting the link between parenthood and parental mental illness, *TFM* might help to promote children’s wellbeing by supporting their parents and vice versa.

In view of the increasingly subspecialized treatment approach, the challenges with including FFP in line with national laws and regulations, it is particularly important to explore how clinicians perceive the implementation of a broader family focused approach. While there is increasing research examining clinicians’ experience when implementing other models to assist FFP, (i.e., Lets Talk), in adult mental health services (11, 26, 27), to the best of our knowledge, there is limited research on clinicians´ experiences with implementing *TFM* parallel in *both* adult mental health and children’s services (39).

The aim of the study was thus to explore clinicians´ experience in implementing *TFM* in both CAMHS and AMH services in a Swedish context. Furthermore, we wanted to gain knowledge of how health care professionals experienced the process of implementing this specific model, regarding individual, relational, and organizational aspects.

## Material and methods

This qualitative study had a descriptive, exploratory design, which was considered appropriate to acquire a deepening understanding of the participants’ experiences with the implementation of a new model (40–42).

### Context

In Sweden CAMHS and AMHS are distinct and different services and in the Stockholm region some services are run in public healthcare while others are run privately. In this study we included public CAMHS and private AMHS. The region serves about two million inhabitants and the services that initially implemented *TFM* serves approximately 170,000 inhabitants. Literature suggests that within CAMHS experienced clinicians are often more family focused while younger clinicians are more accustomed to working individually and the wider organization is more focused on individual maps for care providing (43). In AMHS, care has developed during the last 15 years from less of an individual to more of a family focus; particularly on children as next of kin (44). The AMPHS in this project has over the past decades, systematically and with comprehensive management support, shown an interest in the implementation of a variety of FFP interventions, including Beardslee’s Family Intervention (45–47).

### Ethical considerations

The study was initiated and financed by CAMHS and AMHS and The Swedish National Board of Health and Welfare. The study was assessed and approved by Heads of Operations and the Swedish Ethical Review Authority Dnr: 2023-04618-01. The study is based upon a service development project within the framework of regular quality enhancement. All participants gave an informed, written consent to participate. Data were transcribed anonymously, by removing or altering possible identifying details. The procedures in the study have been made and implemented in accordance with the Declaration of Helsinki (48) and has followed the Swedish Ethical Review Act (49).

### Participant inclusion and exclusion criteria and recruitment

Inclusion criteria were that participants had to have undertaken a two-day training in the model which included web-training and face to face discussion and supervision in the model and that the participant’s managers and service setting were also included in the project. In total 60 clinicians were trained in the model in groups of approximately 15 people from both CAMHS and AMHS. Participants also had to understand and speak Swedish language and be permanent employees in their organizations. Services includes a total of approximately, 300 employees in the CAMHS and 300 in AMHS services. All the 60 trained clinicians had the same training in the model, and each clinician had the possibility to use or not to use the model in clinical practice. Further, the participants were selected based on convenient sampling where their accessibility and desire to participate, in consultation with service and unit managers, guided the recruitment and with the aim of obtaining feedback from a broad range of clinicians.

### Data collection

Focus group interviews were conducted to afford participants an opportunity to elaborate and discuss a variety of experiences and reflections around the implementation and use of *TFM*. Three preplanned focus group interviews (50) were carried out with a heterogeneous sample of clinicians including some with managerial responsibilities, some of whom had used *TFM* following training, and some who had chosen not to. Focus group interviews were caried out separately with clinicians who decided to use the model in clinical practice and with those who had chosen not to use the model. Data were collected using a semi-structured interview guide with open-ended questions, allowing the participants to freely share their experiences (For more detail see Appendix 1). The semi-structured interview guide was developed by CL and MÖ together. CL has substantial experience of FFP in clinical practice and MÖ has an extensive experience in applying semi-structured interviews in research. The interview guide was pilot tested with a group of experienced clinicians. The data collection took place in early 2023. Focus group interviews are suitable when aiming to gain more than individual perspectives (50) and may stimulate participants reflections on e.g. habits and complex phenomena such as the health services through dialogue (51). The focus groups were facilitated by the first author. Whenever possible, the date and location were determined by the participants.

### Data analysis

Data were analyzed using Naturalistic Inquiry (42), in which participants’ descriptions of reality was the main focus. Furthermore, the nature of truth statements is viewed as context-bound, with focus on differences in experiences (52). The interviews were transcribed verbatim (CL). The analyses tried to inductively explore and systematize data on the participants’ experiences of implementing and using TFM.

Each focus group interview was reviewed for an overall perspective and reflections noted (CL, MÖ). The scrutinization included an iterative process, where CL and MÖ went back and forth between their interpretations and descriptions in order to revise the descriptions against the original interview data. Both authors read the transcribed material separately and several times, searching for interpretations and meaning units, and further labelled these with codes (53, 54). Codes and recurrent themes were recorded, and parallel mind maps produced. The next step was to organize and re-organize the meaning units into preliminary and final themes, which in the process were discussed (CL, BW, MÖ) until agreement was reached. Finally, three main themes were developed from the data.

To increase the credibility of the analysis, we used triangulation (42), whereby CL and MÖ individually and then collectively analyzed and discussed the results. Further, BW discussed and gave input to the organization of themes and sub themes. Participants in all three focus groups were asked the same questions; however, the order of which could vary depending on the participants’ reflections. At the end of each interview, the participants were asked to elaborate on anything they found relevant, but which had not been discussed. Alongside the analysis, a table was compiled with quotes from the three focus group interviews. The table was used as a tool to compare results between and within the groups.

When the traditional analyses according to principles of Naturalistic inquiry was completed our collective clinical and research expertise allowed the team on a collaborative level to discuss the results on what we called a meta understanding of the result. The traditional analyze provided grounding of understanding the result, whilst the collaborative work allowed a meta understanding of applying the results to a broader domain. Thus, although not common in research exploring mental health issues and interventions, a further process took place among all the authors together, to reflect upon the results and to analyze it from above “as a bird’s eye view”. Within this, the authors adopted a more detached stance to the data analysis, obtaining a supplementary level of distinction above the objective view, with an aim of gleaning more general principles in the data.

### Philosophical underpinning

In this paper we use the term psychiatric illness/services since the study has been carried out in the specialized health services, where those having the mental health challenges are referred to as patients and have got a diagnosis.

In accordance with Bringselius (55), the implementation process for *TFM* was trust-based, taking into consideration, the views of both those doing the implementation and the subjects (family member) participants. Implementation of *TFM* (38, 56) took place in both CAMHS and AMHS. The purpose was to enable clinicians across the service spectrum to talk with family members about the impact of mental illness on family life and on each family member (not just the index person). This collaborative approach allows for exploration of individual and collective strengths and vulnerabilities and what each person might be able to do to improve their situation. Moreover, we took an experiential orientation to data interpretation which allowed to prioritize professionals own accounts of their experiences and perceptions. This approach also includes the opportunity to reflect on family relationships, communication, and shared understanding.

### Position within the data

CL, a PhD student, had extensive experience of working alongside mental health professionals, in both CAMHS and AMHS as well as MÖ, with an substantial experience of interviewing healthcare professionals (e.g., [5, 39]) and longstanding research concerning family aspects around the person with mental illness. BW was a senior researcher in mental health and inclusivity, whose research has examined family models. AG was a senior lecturer, with expertise in caring science and family focus practice. AF, senior psychiatrist with extensive clinical experience of mental health patients in both AMHS and CAMHS and source of developing the *family model*.

### Reflexive judgement

Authors met regularly to discuss sampling, recruitment methods, coding and theme development. The diversity within the author team with regards to clinical background, experience of working with families in mental health services and experience of conducting qualitative research, allowed us to challenge each other’s assumptions and pre-conceptions when collecting and analyzing the data. Our collective clinical and research expertise allowed the team to focus not only on a basic analysis in accordance with Naturalistic inquiry but also on a secondary analysis of the data we called meta understanding of the result.

## Results

### Participants characteristics

The participants (N=14) all had extensive professional experience in mental health services and had varied professional backgrounds (i.e., social workers, nurses, psychologists, and other treatment staff) and worked in various services, including CAMHS, AMHS, and adult addiction treatment services. Five managers, who had used the model in clinical practice in these three services, participated in Focus Group 1, six mental health professionals who had used the model participated in Focus Group 2 and three mental health professionals who actively chose not to use the model participated in Focus Group 3. Seven of the clinicians were employed in CAMHS, five came from general psychiatry in AMHS and two came from addiction treatment in AMHS. The conversation with each focus group had an average duration of 50 minutes. Participant’s demographic details are detailed in **Table 1**.

**Table 1:** Background information about interviewees.

Number	Participant	Organization/Roll	Experience	Trained/used TFM
		*Managers*		
1.	women	AMHS	Experienced	Yes, >10 times
2.	women	AMHS/dependence	Experienced	Yes, not used
3.	women	CAMHS	Experienced	Yes, >10 times
4.	women	CAMHS	Experienced	Yes, not used
5.	man	CAMHS	Experienced	No, not used
		*Clinics, used TFM*		
6.	women	CAMHS	Experienced	Yes, >10 times
7.	women	CAMHS	Experienced	Yes, 5-10 times
8.	women	AMHS	Experienced	Yes, >10 times
9.	women	AMHS	5 years	Yes, >10 times
10.	man	AMHS	Experienced	Yes, >10 times
11.	women	AMHS/dependence	Experienced	Yes, 5-10 times
		Clinics, not used *TFM*		
12.	man	CAMHS	Experienced	Yes, not used
13.	man	CAMHS	4 years	Yes, not used
14.	man	AMHS	6 years	Yes, not used
	14 total	7 AMHS+7 CAMHS		13 trained of 14 9 active users

Results are reported in two parts. The first part focuses on findings pertaining to use of *TFM* in services and the second part provides a meta understanding of the clinicians’ experiences.

Most participants reported that *TFM* contributed different insights during the process of using it, which appears to have influenced both their individual treatment approach and the broader clinical practice approach in each team/organization.

Results of participant feedback are presented in three main themes: individual, relational and organizational. Feedback from participants in CAMHS and AMHS was compared and contrasted in the latter theme. These themes were further subdivided into categories to illustrate in more detail how clinicians/health personnel experience the process of implementing *TFM*. Individual participant quotes have a numerical prefix. Clinicians and managers are represented with a C and M respectively. Those with both manager and clinician experience are prefixed C/M.

### Main theme 1: individual

*The experience of embracing TFM and ‘having a go’.*

The three sub-themes; ´sceptical´, `more and more adept´ and `why have we not used it before? ` showed that working with more than one person in a family context was a significantly different and often challenging prospect for the individuals’ participants in most mental health services. Also, the experienced clinicians used to work with families found a novel family focused approach as unnecessary, irrelevant or time wasting. Clinicians regardless of their level of experience, experienced multiple barriers to adopting a novel family focused approach.

The participants elucidated the importance to understand the clinicians´ own decision to participate voluntarily in a process to learn about and use a novel model to enhance family focused skills. The participants´ experiences of multiple acknowledged barriers and at the individual, team, organization level and the health care system is designed to focus on the individual service user/patient impede the individual adopting a new model.

#### Sub-theme 1.1: scepticism

*The Family Model* was introduced during a period in which clinicians in CAMHS felt systemic family therapy experienced a failure to continue. Participants who chose not to use *TFM* after training provided this as a reason not to use it. Some clinicians were disappointed that their organization was now showing an interest in the family perspective given that previously functioning structures with therapeutic family networks had been abandoned. “*The model was received with a sigh … I have to say it felt a bit like old wine in a new bottle, so I had a hard time rustling up any enthusiasm”* (12 *C*). Some feared that *TFM* approach would reduce clinical activity rates and lead to concerns “*dirty looks*” (10 C) from their managers.

In some cases, family therapists in CAMHS reported that the organization had long-standing treatment models that were not family focused and instead advocated for individual assessments and “*according to form-thinking*” (12 C) to handle relevant diagnoses. “*We have worked a lot on … specifically with a focus on interventions aimed at diagnosis and very little on the family perspective … which has then ended up taking a back seat according to family focused treatment”* (3 C/M). Despite this resignation, however, it appears that participants not even using the model believe that… “*the approach is in the right place, the systemic focus needs to be kept alive*” (12 C). “*I have to say, when it was introduced to different units it felt really positive, specifically to avoid losing the systematic family focused approach*” (5 M).

#### Sub-theme 1.2: one becomes more and more adept

Several participants who used *TFM* described feeling initially uncertain about how to use it in clinical practice; however, after only a few conversations with families, they reported feeling more confident. One participant described their experience as “*it gets progressively better; you never know what could turn up,, better to feel your way forward”* (8 *C*). Others described *“You get more and more adept*” (6 C), “*Yes, it fulfils a need as we have a clear responsibility to always check if there are children in the family, how they feel and how they can access help, it is very important. The Family Model formalizes the ways to explore this*.” (10 C).

Another participant stated… *“the more Family Models you have carried out, the easier it is to trust the model. At first, I thought you would need to incorporate specific questions due to it being so broad in nature, but it is not necessary*” (9 C).

Several participants in CAMHS reported that it is good that the model works for a broad target group of clinicians and that it is easy to use. It was described as *“a tool that everyone can use, you do not have to be a family therapist”* (6 *C*). For clinicians who habitually work with families and were used to working holistically and with parents as well as applying systemic thinking, the model is considered “*simple and self-evident*” (6 C).

#### Sub-theme 1.3: why have we not done this before?

Participants described how the organization already had several interventions to offer families, but that it is was not until they started using and implementing *TFM* that they realized it contributes something beyond that of any previous family focused intervention.

“*Contributes something I did not know I needed”* (1 C/M).

Experienced clinicians described that to their surprise, that they had not recognized the value of applying a model like *TFM* previously in their professional careers. At first, many thought the broad approach of the model was unfamiliar and challenging. They felt it was challenging to know what to do with all the information, that they were unable to attend to everything. However, it was the families who were able to set a range of processes in motion. They described how the model expanded the family perspective so that everyone in the family, adults as well as children, can participate on more equal terms and on the basis of each person’s perspective and abilities, *“The family system become invigorate by focusing on resources in the family”* (11 *C*). Sometimes, the family continued this work at home in preparation for future sessions. This experience, together with training in the model, contributed to the clinician’s perception that the model was relevant and valuable for supporting service users and families *“The Family Model has initiated a valuable discussion about family focused clinical practice” (14 C)* Another participant expresses *“…the need to think a little more broadly, to look at the context these patients live in?…That is the most obvious impact I have observed” (13 C).* Or as one participant expressed *it “Several of my colleges have told me why haven´t we done this before”* (7 C).

### Main theme 2: relational

The most potent clinician adoption/facilitation appeared to occur when direct conversations could be hold with family members. The participants experienced that the family members brought a different perspective, could be an asset in therapeutic work and clinicians could see (sometimes after a single conversation) how powerful this broader approach could be. Further, there were description of that they found it richly rewarding to have a collaborative conversation with family members in which everyone’s voice was heard and that the process was appreciated by the family. Three sub-themes made up this main theme relation; ´The mess become structured´, `The Family Model throws light on the situation of siblings` and `Healthcare systems may resist change, but families do not.

#### Sub-theme 2.1: the mess becomes structured

Experienced participants in both (CAMHS and AMHS) services described the model as helping to provide an overview and structure in what families initially perceive to be chaotic and messy. *“It provides the ability to map and see how The Family Model throws light on the situation of sibling to move forward with interventions that enable us to see the aa whole family’s situation. I think it really highlights a need, an established need”* (3 M/C). The model was also described as structured and easy to use, “*this map makes it easier for me to address this and to reflect on it” (8 C).* Conversations related to strengthening the role of the parent, became easier for the clinicians to manage … *“is perceived as a support in strengthening the role of the parent” (11 C).*

Participants also described that they felt that, through using the model, families were better able to see who does what (role clarification), allowing for more realistic expectations, as well as becoming more aware of the help available from external sources several parties. Perspectives were broadened for both clinicians and families, *“Above all, you get an overview, a sort of visual model* (2 M/C).

Participants reported the usefulness for the family and the participant clinicians *“There is a lot to keep track of when children and parents each have their own healthcare providers. It is often like a full-time job for these parents to try to manage the extensive healthcare system around them…”* (9 *C*). *To get the whole picture … well, people are usually pretty relieved … yes, that is it, that is what it is like.”* (6 C). Participants revealed how the families gained confidence through their ability to manage both resources and problems thanks to the structured design of *TFM*. They expressed that this often provided valuable insight for both clinicians and family members, while also pointing out *“all the good things in their family”* (11 *C*).

#### Sub-theme 2.2: the family model throws light on the situation of siblings

Across all participating organizations, clinicians and managers reported that siblings have been neglected, and that their needs are frequently neglected. Some participants reflected on siblings who were minors. One manager especially reported that *TFM* conversation had brought attention to this target group, *“The Family Model has thrown light on a gap in knowledge, namely siblings”* (2 M/C), another described the importance of including siblings as they have a major impact on the unwell child, “*siblings are often factors of health and attention must be given to these … it becomes prevention by using The Family Model”* (6 *C*).

#### Sub-theme 2.3: healthcare systems may resist change, but families do not

Some participants expressed that it was difficult to start using *TFM*, but the realization that it is liked by families led to their continued use of the model. They observed that families did not avoid participating in conversations using *TFM* and the associated changes brought about by the model. This is in great contrast to what was described as clinicians’ own abilities for change: “*We work hard all day to get other people to change behaviors, but we ourselves are firmly stuck in our old ways”* (12 C). Instead, they noticed that families enjoyed being involved in the conversations and participating on equal terms with clinicians in working through the family’s situation, in particular developing a better shared understanding and ‘ownership’ of their challenges and potential solutions: “*they enjoy it, they are personally involved in formulating what needs to be worked on or improved, whatever that may be”* (3 M/C).

Participants described how *TFM* contributed to the development of parents in their parenting roles when they were given the opportunity to listen to their children describe their views, which helped parents see a greater whole. Parents expressed appreciation of being made aware of the thoughts and views of the children, *“…one boy, 12 years old, the eldest of five, was able to accompany his mother and talk…. this way, the parent also got the whole perspective and was completely amazed by her child’s thoughts and views.”* (9 *C*). Clinicians also noted that children wanted to be included themselves in conversations and also wished to include the parent who was ill in the conversation. One clinician recounted a girl’s wishes as reflected in the following quote, *“this was good but my dad who is unwell should have been a part of it.”* (6 *C*). Another clinician indicated*, “I see this as a clear area for improvement”* (7 *C*). Participants described families’ expressed reflections “*Why have you not used this before? We belong together, we are a family*” (6 C). Participants also described how the model contributed to an increased transparency of parent reporting a change of thinking from *“I am such a bad parent”* to being more likely to say, “*this works but some more work needs to be done on this*” (8 C). One participant described *“Something I have noticed is how much confidence is gained by those who see us for treatment when using the model, the growth in their parenting.”* (10 *C*).

### Main theme 3: organization

A sum-up of the participants experiences regarding organization was that they found.

*TFM* clearly to be a tool with utility to support the clinician ‘have a go’, that does not require intensive and lengthy training, is practical and understandable to family members including children. The participants experienced a tool which supports a shared and collaborative conversation with a practical outcome, and easier for service managers and organizational leaders to support in the implementation process. Four sub-themes made up this main theme; ´Something to offer in accordance with their service duties´, ´Synchronizing the (family focused) self-image – the pendulum needs to swing back`, `Needs for a focus on the family perspective within policy documents to promote FFP` and `Not everything is psychiatry.

#### Sub-theme 3.1: something to offer in accordance with their service duties

Participants stated that they already had other interventions to offer families but that these were more resource-intensive and therefore benefitted only a small number of families. Several participants (clinicians and managers) described how positive it felt to have a less resource intensive and simpler model to use, *“it has a low threshold for implementation and can reach many people in contrast to other family interventions”* (1 M/C). One manager described how *TFM “provides an option that fills the gap, easily and clearly”* (2 M/C*)*. Another participant reported that *“everyone feels the model is a good idea and understands the benefits of it. However, there is a threshold we need to cross*” (3 M/C).

In CAMHS, participant managers reported that *TFM* helped to bridge a gap in recognizing the need for family work, something which current guiding systems in the service did not highlight. They reported that… “*the family perspective has not been captured in our guiding systems on a higher degree”* (3 C/M), *“the family perspective needs to be included* (5 M).

*“We see the benefits of the model. It provides us with a direction now and that is timely”* (4 M). As one of the participating clinicians put it, *“it was simple to link the family perspective with the child perspective”* (13 *C*).

Participant clinicians from AMHS stated that they already had other interventions to offer families but that these were more resource intensive and therefore benefitted only a small number of families. Several participants (clinicians and managers) described how positive it felt to have a less resource intensive and simpler model/approach to use, *“it has a low threshold for implementation and can reach many people in contrast to other family interventions”1 C/M.*

#### Sub-theme 3.2: synchronizing the (family focused) self-image – the pendulum needs to swing back

Overall, participants from CAMHS described a loss of previous family focused knowledge and expressed misgivings about incorporating the family perspective into new management models and introducing it to new staff members. One manager expressed it as, *“We earlier had a self-image of being family focused … but have lost the family perspective and need to reset it*” (5 M).

Similarly, AMHS participants described predominance of person-centered treatments. Experienced professionals retained knowledgeable and experience in working with families, but managers reported that particularly recently qualified staff trained in the person-centered era had not been supported in family focused perspectives and practice. They highlighted the need for *“support and a working method that is easily accessible and uniform with regard to more old family dynamics”* (3 M).

It is understood that family, parents, relationships and context affect the child’s situation, but clinicians had not managed to include the family perspective, *“We have a self-image within AMHS about an expanded patient perspective … we need to harness this ability when it comes to parental well-being interacting with the child’s well-being*” (5 M).

In AMHS, participants met many patients who were themselves children of a parent with mental illness and reported how these patients react when they saw accessible information about the model, for example *“if only someone had paid attention to me when my parent was mentally ill, how different everything could have been”* (1 M/C).

#### Sub-theme 3.3: needs for a focus on the family perspective within policy documents to promote family focus practice

In CAMHS, both staff and managers described a lack of reference to families and a family focused approach within policy documents in both service level policy and at a national level. Furthermore, several participants in CAMHS stressed that care support systems had to develop the family perspective *“policy documents need to take the family into account, by highlighting the family’s situation and the importance of the interactions”* (7 *C*).

Participants stressed how family work, when added to policy document in both AMHS and CAMHS could enhance the child perspective *“that family work enhances the child perspective”* (1M/C, 3 M/C, 9 C, 10 C). Clinicians stated that the experience of treatment interventions with the whole family sitting together *“should enhance the child perspective”* (8 *C*).

Clinicians in AMHS perceived that legislation, as well as organizational policies, guidelines and procedures which recommend that professionals should always ask questions about the existence of dependent children in service user’s families would contribute to the streamlined introduction of *TFM* into the organization. *“The structure needs to provide support through policy documents that take the family perspective into account*” (14 C). In addition, participants perceived that established child support persons and child coordinators in the organizations dedicated to highlighting the child perspective contributed to the establishment of implementation of *TFM*. In addiction services, it was observed that clinicians reported that similar structures, even if these had not been in place for as many years, have actively contributed to the introduction of family work and *TFM* use.

In CAMHS, participants reported a need for clearer overarching support in the structure to establish the model, which they indicated could be achieved in different ways. One option is to bring the treatment focus back to a more family focused approach. Another proposed option is to introduce a ‘parental support person’, “CAMHS *needs a parental support person or adult support person to enhance the family perspective*” (3 M/C). In both services participants expressed a desire for the organization to provide increased support for the use of resources required by *TFM*, …*”a level of commitment is required from the organization that confirms that it, the model … is given the time it needs”* (10 *C*), or “*we need support from the employer for the time it requires and for it to be okay to intervene and provide parents with support*” (7 C*).*

#### Sub-theme 3.4: not everything is psychiatry

Participants described that patients and families seeking care had different circumstances and reasons for seeking psychiatric services at a specific point in time. The clinicians expressed how, after implementing *TFM*, they became more aware that psychiatry alone could not do it all for families and children, and that there were other important roles in society and in the family’s own network that can contribute, expressed as “*the model works well as a supplementary or additional tool or for screening purposes … not everything is psychiatry.”* (10 *C*).

Having one or several conversations using the model may also been suitable in the assessment of whether specialist care was the most appropriate. “*I believe The Family Model has an effect on resources distributed to the families and savings in the systems beyond the implementation itself.*” (14 C). The participants perceived that the model helped to distinguish the commitment from psychiatric services when clarifying psychiatric services obligations to the families, and for staff when in need of understanding their health care obligations contra other health care providers. The first situation when participants expressed ending *TFM* session with *“Finally I understand what the mission of child psychiatry is”* (C 13), which gave more sound expectations for both professionals and patients concerning what the services offers and when the mental health services responsibility and opportunity end. But also, with participants experienced the parents` understanding of their role in the family “*We may get a referral that describes multiple problems with the implicit desire for psychiatry to fix this … when you experience the family’s situation more systemically, I can more easily see what treatment is*” (C 11). *“The mapping makes it easier to understand the complexity of expectations and what is durable”* (M/C 1). Some participant described the model’s usefulness in highlighting when psychiatric patients had been in treatment for a long time, more out of habit than out of actual need, “*this is not psychiatry … we can terminate the patient and send a referral to the family doctor for medication follow-up only”* (C 8).

### Meta understanding

Regarding clinicians’ experiences with *TFM*, the meta understanding found ‘that something’ which, at the individual (clinician) level generated the final step in deciding to ‘have a go’, attending the training, doing the (free) eLearning course, talking with the patient/parent or relative about *TFM*, or booking a time and doing that first session. Furthermore, there appeared to have been a decisive moment in the clinical encounter when angst and uncertainty of the new approach was eclipsed by the ‘aha moment’ of enhanced awareness when important communication occurred or when they felt reward/satisfaction after the shared achievement of a collaborative care plan.

**Figure 1:**
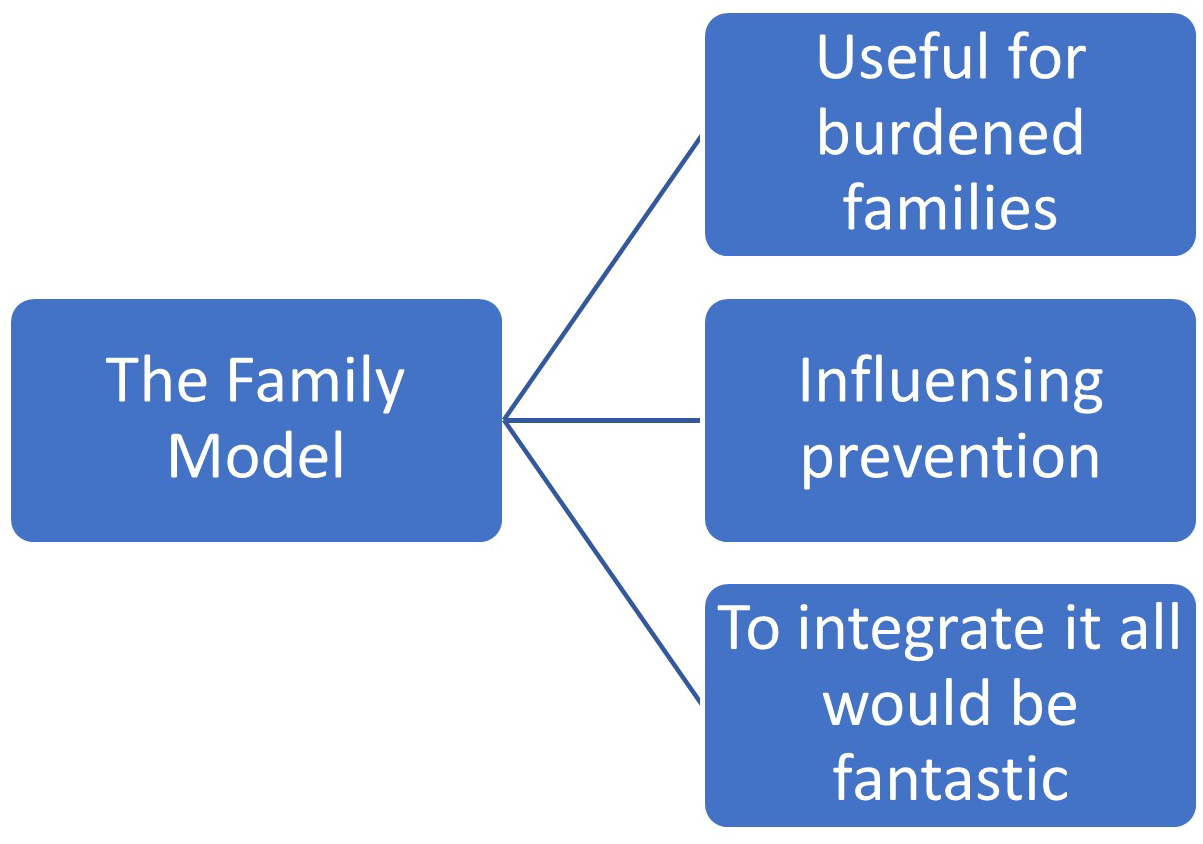
The Family Model from the meta understanding.

Participants suggested that *TFM* is “Useful for burdened families”, “Influencing prevention”, and “To integrate this would be fantastic” (**Figure 1**). These responses depict an overarching understanding of the model, that those with extensive clinical experience discerned and put into comprehensible meaning.

#### Meta theme 1: useful for burdened families, especially while handling privacy legislation

The model was seen to be particularly useful for extensive mental health issues in families with more than one family member experiencing mental illness and with multiple healthcare providers. The model highlighted the needs and abilities of all family members and sees them as a collective group.

Some described the model being used in collaborations between CAMHS and AMHS and viewed it as very effective in these instances. One participant who was yet to start using the model but who had participated when colleagues have used it suggested that it was useful in cases where families were accessing care from several different healthcare providers, “*sitting down with the model in these cases demonstrates the need for collaborative models*” (13 C). Moreover, they described the common training initiative between AMHS and CAMHS in *TFM* contributed to encounters across organizational boundaries and mutual support, despite a previous lack of collaboration of this kind. Regarding this, participants reported that the collaborations initiated by the model made things *“really click*” (6 C), or as someone said, it provided the realization that *“child psychiatry would benefit from having a little adult perspective as well” (12 C).*

Furthermore, the model was described as effortlessly handling privacy legislation given that all family members participated in the discussions as well as in the decisions on the next steps to be taken and possible needs for and forms of continued collaboration, “*a focus on how collaboration can enhance the process, where everyone is brought together based on a focused format, when required by the family”* (10 *C*). Participants also mentioned the applicability of the model when a parent’s involvement/ability could be extended, given that the model at an early stage involved “*sitting with the family who produce a family plan together and decide on priorities to enhance the child perspective”* (4 M). Or expressed in another way, “*it provides the space for the child and relatives to describe their perspectives.”* (9 *C*). The structure of the model meant that all participating family members were heard.

#### Meta theme 2: influencing prevention

Participants, particularly in AMHS, reported that many children with parents experiencing severe mental illness end up requiring adult psychiatric services and addiction care later in life. However, all the interviewed clinicians described a genuine determination from parents to ensure that children received optimal support to prevent this progression regardless of whether they were CAMHS patients or children of parents with mental illness. The model was described as an opportunity to contribute to “*one way to prevent the progression”* (2 M/C) from CAMHS to addiction care or AMHS.

#### Meta theme 3: to integrate this would be fantastic

When participants reflected on the future, they stated that the model could contribute to *“Bridging the gaps between CAMHS and AMHS”* (5 M) *and “linking arms with each other”* (12 *C*), something that was increasingly sought by clinicians in the different organizations.

Using the model as support, this process was seen to be achievable. Bridging the gaps between CAMHS and AMHS was seen to contribute to families being given more control over the care process as well as when and which kind of care or different forms of intervention were best provided. An increased sense among parents of having a clear grasp of the situation is described as necessary; participants reported how exhausting it was for parents to have multiple different healthcare providers, especially when it comes to the healthcare needs of children. Participants described how parents currently accessing CAMHS who also suffered from mental illness and associated difficulties would be supported by closer relations between CAMHS and AMHS, as a more coherent picture of the family situation would make it easier for both families and clinicians, “*to integrate that more would be fantastic”* (2 M, 4 M).

## Discussion

**Figure 2:**
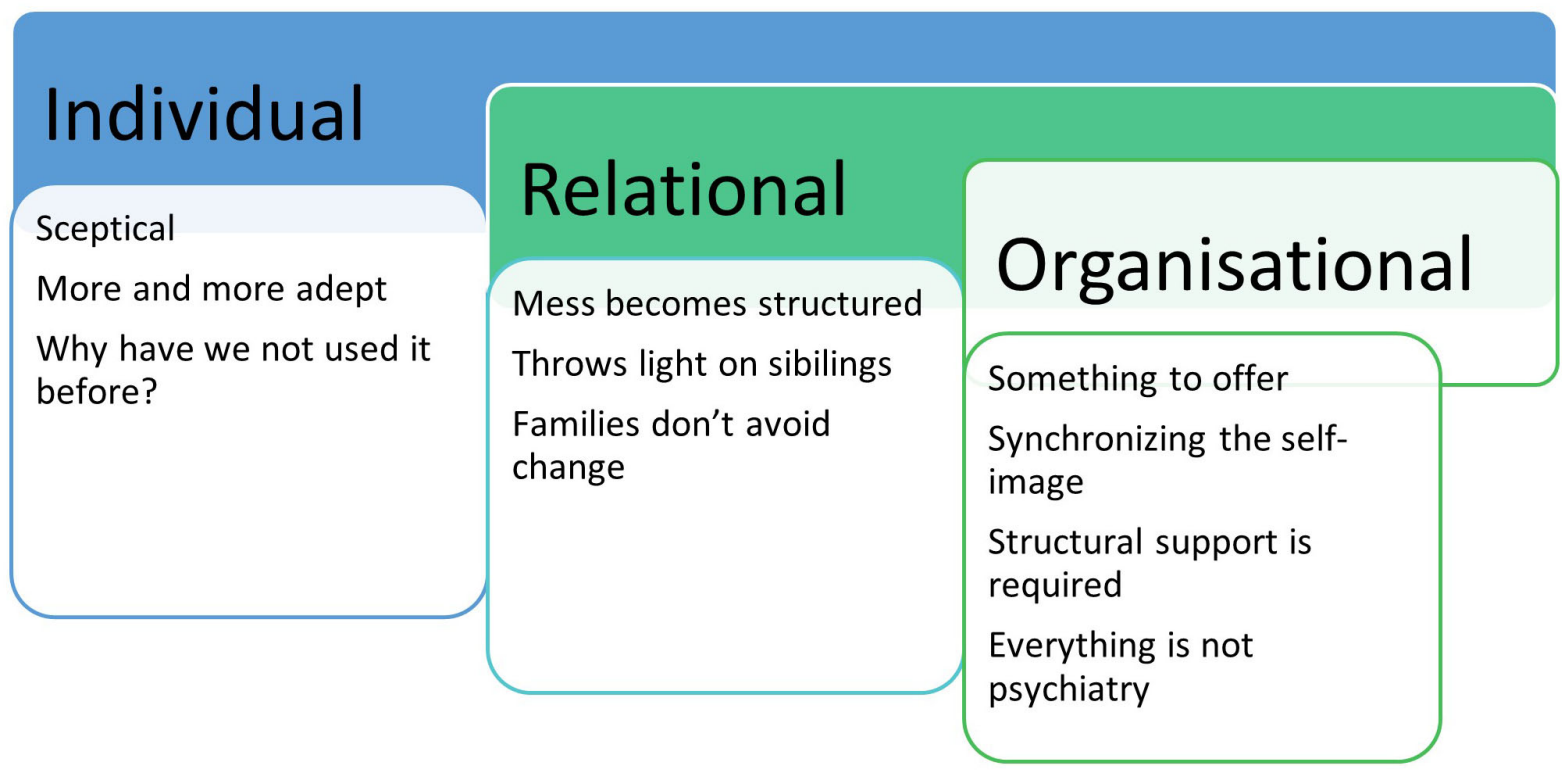
Conceptual Summary of key themes.

This qualitative study described participant (CAMHS and AMHS clinicians and managers) experiences using *TFM* as part of regular care and treatment of their patients and families. Their feedback suggested that *TFM* has been a useful tool to support family focus practice. Their reports indicated changed views/experiences as the implementation process progressed, from initial scepticism to acceptance as they engaged with and used the approach, with some expressed surprise that this approach had not been introduced earlier (see **Figure 2** – Individual).

Feedback also highlighted the benefits of relational aspects when clinician interacted with family members, particularly useful of acquiring a broader understanding of the index person’s circumstances and context (see **Figure 2** – Relational). For example, ability to manage complex needs (more than one person with MH challenges) and awareness of sibling needs. Although speculative, it is possible that the simplicity of *TFM* approach guided by its visual structure assisted family members’ understanding and supported clinicians in making sense of an individual’s needs within their family context.

### Clinicians surprised by the enthusiasm shown by families for TFM

Perhaps of most significance was the positive response from family members when introduced to *TFM*. Participants described their surprise at the enthusiasm with which *TFM* was received by families, including families with multiple members experiencing mental health challenges. Clinicians described how (within the framework of *TFM*) families independently took the initiative to solve problems (empowering), and how the model contributed to the family being aware of areas that were working well within the family (focus on strengths).

### Clinicians positively influenced by family members’ uptake of TFM

This enthusiastic uptake by some families appeared to have motivated their clinicians (who were perhaps still in their ‘sceptical phase’), indicating that in some situations family members were less resistant to change than their clinicians. It also illustrates how *TFM* can empower family members and assist them to ‘find their voice’. Patient/family members’ influence on clinician behavior regarding FFP has not often described but their influence was reported to be an important aspect in the clinical engagement process and should be more emphasized than it has been in the past. These results are in line with previous research (19, 57–59) demonstrating the value of family members needing to feel supported to better assist their unwell relatives when they experience mental illness, and that support for parental mental illness benefits the whole family.

### Use in AMHS and CAMHS

There were participants from both CAMHs and AMHs, suggesting *TFM* utility across the age spectrum of service/clinical need and the universal relevance of FFP (see **Figure 2** – something to offer). Participants in both services found *TFM* clinically beneficial for working with the patient in a more family focused way. Furthermore, it is possible that the simplicity in concept and practice of *TFM* made it easier for managers/leaders to support clinicians in the implementation process at a lower threshold and reaching more families.

### Links between CAMHS and AMHS

Some participants commented on how their use of *TFM* supported cross-service links between AMHS and CAMHS, often for the first time. The use of *TFM* created opportunities for improved communication, less duplication of effort and roles, and better care coordination. In addition to improved cooperation between AMHS and CAMHS in Sweden, our result concerning increased cooperation might influence other countries that already use the model. Despite differences in health service structures, funding, policies and training, clinicians, managers, and family members were able to understand and use *TFM* as a useful conceptual framework and as an entry level intervention to assist practitioners in implementing their FFP. Furthermore, participants reported no language barriers in understanding or using *TFM* approach.

### Meta understanding: what motivates clinicians and managers to use TFM?

The participants experienced that their decisive/defining moment in the clinical encounter, when angst and uncertainty of the new approach was eclipsed by the ‘aha moment’, occurred when important communication appeared. They told of moments when they attended training, talking with patients´ about *TFM*, and booking a time and doing a first session.

They expressed satisfaction after the shared achievement of family members gave positive feedback about *TFM* conversation (see **Figure 1**). Participants with extensive clinical experience clearly described their views about *TFM* as “useful for burdened families”, able to help with “Influencing prevention”, and they expressed enthusiasm for implementing the Model: “To integrate this would be fantastic”.

### Clinician experience

*TFM* was perceived as particularly user-friendly among clinicians with previous training in family work, while recently educated clinicians, trained in a more person-centered era, found it more difficult to independently apply the model, especially in the initial stages. These findings are consistent with a review by Fixen et al. (60) showing that the effectiveness of implementing new models depended on user knowledge of the core components, in our case experience of meeting/involving relatives and family members in the clinical work and care plan development. However, once the clinicians, regardless of previous experience, used the model with a number of families, they described how it helped them gain further insight into the family’s overall situation.

### Implications for implementation of FFP – trust- based approach

As well as leadership, addressing the prevailing culture of individualism will require multiple efforts across all tiers of an organization with family focused policy, training, and education (3).

From the starting point of the implementation process for *TFM*, Trust Based Practice (61) was the approach used, since it was important within the implementation process to give confidence to both clinicians’ ability to influence and provide users with better services.

*TFM* appeared to have facilitated trust-based management for focus group participants by enabling them to ‘have a go’, thereby challenging the prevailing culture of individualism and supporting a broader approach to practice (allowing for FFP to occur), by incorporating more than the individual/index patient and more than the core symptom profile/diagnosis. CAMHS and AMHS clinicians supported a view that the current culture of individualism in mental health services had to be complemented with more family focused services, as shown by Leonard et al. (62). This need of more family focus was the case even in instances when the clinician had not personally used the model in their clinical practice.

### Organizational support and timing facilitate a willingness to change

Acquiring new skills voluntarily required clinicians to acknowledge a knowledge gap in the area of focus, to tolerate uncertainty associated with ‘not knowing’ and to overcome the various internal and external structural and cultural barriers in the team/service/broader organization (51). Increasing specialization in education and training allowed/supported clinicians to become increasingly skilled in a more narrowly defined area of practice. This approach has advantages but can also reduce capacity/preparedness to move beyond the known skill set comfort zone, for example, the increasing specialization in mental health for diagnosis specific treatments targeting individuals, hence the increasing challenges for developing and enhancing FFP in mental health services. Clinicians and managers spoke about their use of *TFM* fitting in well with their daily work and scope of practice and that a parallel implementation of the model in CAMHS and AMHS (i.e., implementation across both services) might be a useful way to scale up, providing both intra- and inter-service benefits. Participants also suggested that this parallel implementation process could also support collaborative practice and a shared understanding of roles, tasks, and resources between the services, which should be taken into consideration regarding further research on implementation.

### Proposed actions for organizations interested in implementing *the family model*

- Complete implementation of a new model in an organization usually takes 4-6 years. However, the result had shown that it is possible to gradually implement *TFM* following training and clinical trials from as early as after the completion of testing the model with 3-5 families.
- *The Family Model* provides opportunities for long-term application right from the start and provides support for a decision to maintain it as part of ordinary activities (sustainability).
- Attention should be paid to the value of ‘champions’ to enhance the family perspective within the organizations.
- To ensure fidelity to the model, sufficient time and resources must be allocated to training and guidance in the use of *The Family Model*.
- Use of *The Family Model* can contribute to good and efficient utilization of resources in daily clinical work.

### Strengths and limitations

This project was developed based on a need to introduce a new family focused model which came from the health care professionals themselves. It was further developed in close collaboration with clinicians and users and the head of CAMHS and AMHS was involved in the conditions for starting the change process which can be seen as a strength. Moreover, an established model was selected (focus group interviews) to investigate the experiences of clinicians and managers in the implementation of *TFM*. Focus group interviews had previously been shown to work well with small groups of people who knew each other (63). On the other hand, to include some individual interviews could have strengthened the material, because some perspectives might not be revealed in an open setting such as in a focus group interview. Furthermore, the focus group leader as a part of one of the services included in the project might result in both advantages and disadvantages in analyzing the material.

Moreover, we used triangulation of the data during analysis, which has shown to increase the credibility of the analysis of results (42, 64). The paper might have potential for bias in the analysis concerning the first author also being the focus group leader. It was thus important to discuss the interpretation of the data material openly in the research group. Furthermore, the last author who took an active part in the analysis did not participate in the initial training of *TFM* to maintain an open attitude towards the model and its implementation.

### Conclusions

*TFM* appears to have utility as a useful conceptual framework to support clinician awareness and thinking about family focused practice, and as an entry-level intervention to assist practitioners in implementing their family focused practice. It works to implement the model parallel in both CAMH and AMHS. *The Family Model*, according to clinicians and manager interviewed, influenced resources in the families as well as the services in a positive way. Furthermore, the clinicians interviewed were really surprised by the enthusiasm shown by families for *TFM*, who adapted and used the model as their own communication tool. A challenge to the status quo of existing services and practice does require time, effort, and resources to facilitate implementation of *TFM*. Nevertheless, this requirement could be seen as a relatively cost effective approach to family focused practice.

## Data availability statement

The raw data supporting the conclusions of this article will be made available by the authors, without undue reservation.

## Author contributions

CL: Writing – original draft, Conceptualization, Formal Analysis, Funding acquisition, Investigation, Project administration. AG: Supervision, Writing – review & editing. BW: Supervision, Validation, Writing – review & editing. AF: Conceptualization, Supervision, Validation, Writing – review & editing. MÖ: Conceptualization, Data curation, Formal Analysis, Investigation, Methodology, Supervision, Writing – original draft, Writing – review & editing.

## Appendix 1

### Interview guide – Focus group

1. Does the Family Model meet any need? If so, what?
2. How has the Family Model been used in everyday clinical practice?
3. How has it been received?
4. Is the Family Model suitable for your target group?
5. Has the family model influenced the treatment approach towards a clearer family perspective?
6. Has the Family Model strengthened the child’s perspective?
7. Has the Family Model created better collaboration/coordination in families where parents as well as children has mental illness?
